# Recurrent Intrathoracic Locking of the Scapula after Lung Cancer Resection and Combined Rib Resection

**DOI:** 10.1155/2017/8486739

**Published:** 2017-03-01

**Authors:** Akinori Kimura, Hideyuki Sasanuma, Takashi Ajiki, Hitoshi Sekiya, Katsushi Takeshita

**Affiliations:** Department of Orthopaedic Surgery, Jichi Medical University, 3311-1 Yakushiji, Shimotsuke, Tochigi 329-0438, Japan

## Abstract

We report a case of recurrent locking of the scapula in the thorax after combined lobectomy and thoracic wall resection for advanced lung cancer. The patient was a 52-year-old man with advanced spindle cell carcinoma in his right lung. He had undergone right lung lobectomy and thoracic wall excision (Th1–5). Intrathoracic repair had not been performed to address the defect in the thoracic wall. Two months after the operation he experienced sudden acute pain in the right shoulder. Three-dimensional computed tomography revealed locking of the scapula intrathoracically. The diagnosis was recurrent locking of the scapula in the thorax. He underwent conservative treatment. Because his symptoms were not alleviated and he continued to experience recurrent locking, we performed partial resection of the inferior part of the scapula. Although scapular locking diminished after this procedure, there were still some pain and “catching” between the scapula and the thoracic wall (T6) when he undertook certain movements. No further surgery could be performed, however, because the cancer from the primary lesion had recurred near the previously operated thoracic wall. A procedure for recurrent intrathoracic locking of the scapula was not successful in this case.

## 1. Introduction

Fourteen cases have previously been reported of locked scapula, in which the scapula becomes caught inside the ribcage. In seven of these cases in which patients had suffered high-energy traumatic injury, locking occurred only once and improved with conservative treatment. The other seven concerned nontraumatic injury, in which patients had undergone malignant tumor resection and combined chest wall resection. Five of these were treated conservatively and only two were treated surgically. Patients who were treated conservatively experienced locking only once, whereas those who were treated with surgery underwent recurrent locking. Here we report the case of a patient who was surgically treated after suffering from repeated locking when the scapula became caught within the thorax after chest wall resection to remove lung cancer.

## 2. Case Report

**Figure 1:**
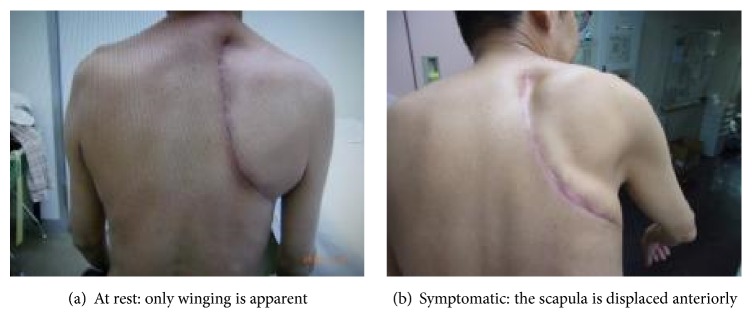
After lung cancer surgery (right thoracotomy).

**Figure 2:**
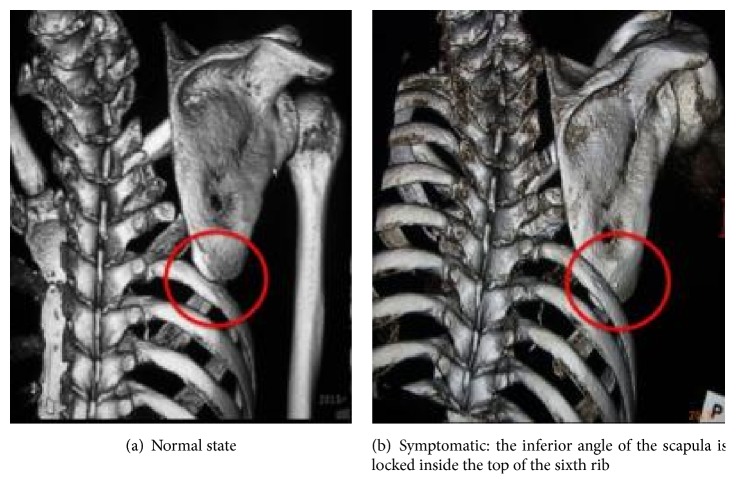
Preoperative CT.

A 52-year-old man who was employed at a titanium-machining factory and who had a history of cancer of the right lung (spindle cell carcinoma, clinical stage T3N0M0) underwent right upper lobectomy and extended combined resection of the chest wall (resection of the 1st to the 5th ribs, right thoracotomy) to remove lung cancer. Chest wall reconstruction was not performed during this procedure on account of the judgment of the thoracic surgeon. He was discharged from the hospital without complications. One week after discharge, sudden pain appeared in his right shoulder during actions such as rolling over in bed, getting up, washing his face, or driving a car, and he was referred to our department with suspected dislocation of the shoulder. Physical examination revealed no resting pain and no restricted range of motion of the shoulder, with only winging present at rest. After the patient performed a push-up movement, however, the pain appeared, and the shoulder girdle was locked at a horizontal abduction angle of 45° (Figure 1). Computed tomography (CT) revealed that when the symptoms appeared, the inferior angle of the scapula was caught inside the top of the sixth rib. This locking was not evident on CT when the pain was absent (Figure 2).

On the basis of these findings, recurrent intrathoracic locking of the scapula was diagnosed. Conservative management was implemented in combination with physiotherapy, using of a clavicle band to restrict the movement of the scapula. However, because there was no improvement in either the locking symptoms or pain, it was decided to adopt a surgical approach.

**Figure 3:**
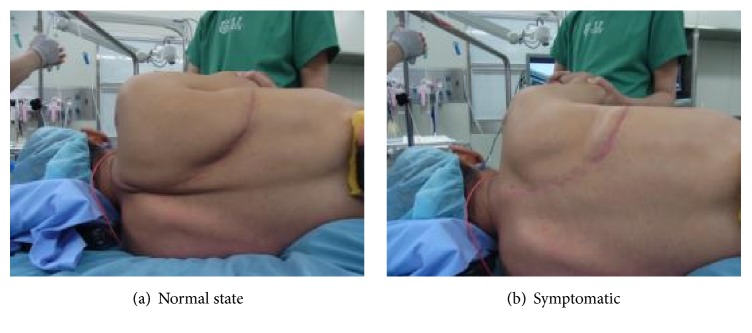
Preoperative positioning.

**Figure 4:**
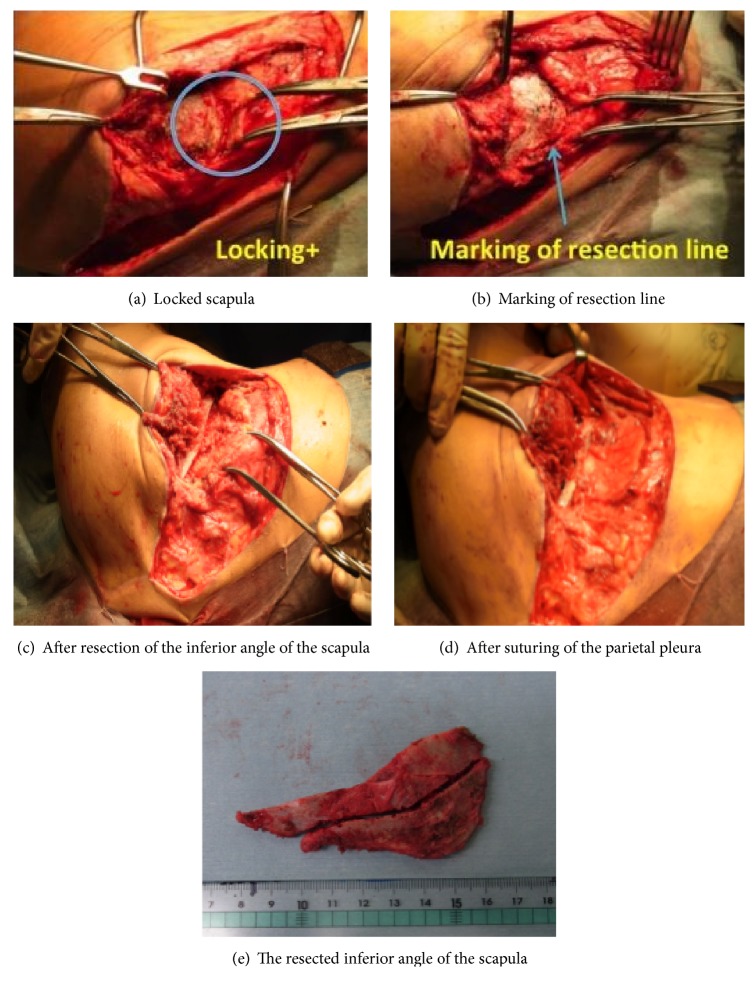
Intraoperative photographs.

Surgery was carried out via a high posterolateral incision (using the skin incision made during chest surgery) with the patient in the lateral decubitus position (Figure 3). The teres major and rhomboid major muscles on the dorsal surface were detached from the periosteum to expose the bone surface of the scapula. The serratus anterior muscle, which had been conserved during the previous surgery, was also detached to enable the inferior angle of the scapula to be raised sufficiently, after which the inferior angle of the scapula was locked inside the thorax and marked with an electric scalpel. A bone saw was then used to remove the inferior angle of the scapula, leaving a margin of +1 cm (Figure 4). A hole was made in the osteotomy section by using a 2.0 mm K-wire, and the teres major and rhomboid major muscles were secured to the chest wall.

**Figure 5:**
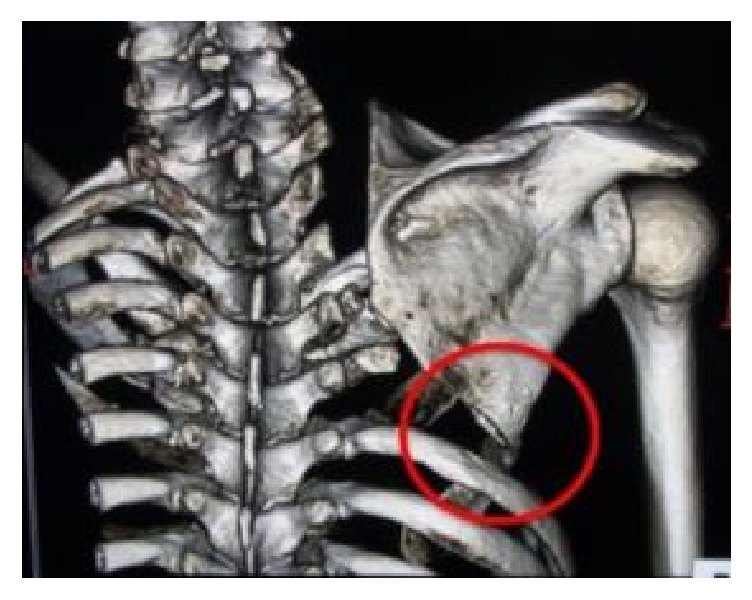
Postoperative CT: locking symptoms improved after resection of the inferior angle of the scapula.

Postoperatively, the locking symptoms improved, but pain still occurred when the resection margin collided with the ribs (Figure 5). There was no change in the range of motion from before. Although another operation was considered, the decision was made to implement palliative care because localized recurrence of the lung cancer was present and metastases had enlarged. The patient died ten months after surgery.

## 3. Discussion

In 7 of 14 reported cases of locked scapula, the scapula became caught in the ribcage after combined malignant tumor and chest wall resection. Five of these patients recovered with conservative treatment, but 2 cases were treated with scapulothoracic arthrodesis [1–4].

Depending on the extent of the malignant tumor, adequate oncologic resection can result in partial- or full-thickness thoracic wall defects. It is widely accepted that defects exceeding more than four ribs at the lateral chest wall are associated with higher risks of herniation and paradoxical breathing and therefore should additionally be reconstructed with a musculocutaneous flap combined with synthetic nets or titanium implants [5–7]. However, the closer the defect to the apex of the thoracic wall, the greater the suspension provided by the sternum, scapula, and clavicle, and even larger defects might be reconstructed without additional synthetic materials [8].

In the present case, chest wall reconstruction was not performed; as a result, the scapula had become trapped within the chest wall. At the primary operation, this preventive procedure should have been performed, considering the risk of recurrent intrathoracic locking of the scapula.

Performing scapulothoracic arthrodesis in this case seemed to be impossible, because of the difficulty in fusing the scapula to the margin of the residual ribs (Th1–5). Additionally, this procedure has been reported to have postoperative complications such as restriction of range of motion, persistence of pain, and prolonged immobilization [9].

There have been no previous reports of resection of the inferior angle of the scapula or partial costectomy. In the present case, given the patient's survival prognosis, our priority was to enable his rapid return to normal life, and we therefore removed the inferior angle of the scapula. However, because we could not estimate postoperative scapular motion during the operation, catching symptoms caused by the 6th rib and residual scapula persisted.

## 4. Conclusion

Recurrent intrathoracic locking of the scapula is a rare complication of rib resection for thoracic malignant neoplasms. Simple resection of the inferior angle of the scapula may not be an acceptable treatment option for scapular instability due to a chest wall defect.
